# Open synovectomy treatment for intra- and extraarticular localized pigmented villonodular synovitis of the knee: a case report

**DOI:** 10.1186/s12891-020-03895-x

**Published:** 2021-01-07

**Authors:** Daoliang Xu, Jianxia Wen, Shisi Zhang, Xiaoyun Pan

**Affiliations:** 1 Department of Orthopaedic Surgery, Second Affiliated Hospital and Yuying Children’s Hospital of Wenzhou Medical University, 109 Xueyuan Xi Road, 325027 Wenzhou, Zhejiang China; 2 Department of Gastrointestinal Surgery, The First Affiliated Hospital of Wenzhou Medical University, Wenzhou, Zhejiang China

**Keywords:** Case report, Pigmented villonodular synovitis, Intraarticular, Extraarticular, Synovectomy

## Abstract

**Background:**

Pigmented villonodular synovitis (PVNS) is a rare, benign, proliferative neoplastic process that commonly affects synovial-lined anatomic spaces. The diffuse type (DPVNS) is characterized by invasion of the entire joint synovium, while the localized type (LPVNS) is characterized by a relatively normal synovial appearance. This report describes a unique case of massive intraarticular LPVNS with an extraarticular extension through the lateral patellar retinaculum. No similar cases have been found in the literature.

**Case presentation:**

A 58-year-old woman had a history of hyperuricemia and knee trauma and presented with unilateral knee acute swelling and pain symptoms with sudden onset. Recent expansion of the LPVNS caused the development of a tender palpable soft tissue mass in the anterolateral aspect of the knee and acute reduced mobility. Preoperative magnetic resonance imaging of the knee revealed the presence of only the soft tissue mass and mild degenerative changes. Open synovectomy was performed successfully to excise the mass. Intraoperatively, macroscopic features of the bright brown inflamed synovium suggested LPVNS, which was confirmed histopathologically. Postoperatively, the symptoms of limited mobility and pain were appreciably relieved. Recurrence was not observed during the clinical follow-up at 1, 6 or 18 months after surgery.

**Conclusions:**

Here, we report the unique case of localized pigmented villonodular synovitis of the knee in a misdiagnosed patient with intra- and extraarticular lesion, which might be attributed to the history of knee trauma and the focal defect of the lateral patellar retinaculum. Open synovectomy effectively relieved the symptoms of limited mobility and pain and no recurrence was observed prior to 18 months postoperatively. To reduce misdiagnosis, MRI examinations are recommended for all patients suspected of having PVNS, including those who have a history of hyperuricemia.

## Background

Pigmented villonodular synovitis is a rare benign tumour of the synovium, characterized by the presence of synovial proliferation and haemosiderin deposition within synovial-lined anatomic spaces [1, 2]. As PVNS is a monoarticular process, it mainly affects the knee (~ 70% of cases), followed by the hip (~ 20% of cases), ankle, shoulder, and elbow [3–5]; in these joints, PVNS causes joint swelling, pain, and stiffness. Individuals in the third or fourth decade of life appear to be more susceptible to this aggressive disease. The annual incidence of PVNS is estimated of 2 – 8 cases per million in the general population with male predominance, although some studies have shown there are no differences between gender [6–10]. Without treatment, PVNS can cause severe degenerative changes and osteoarthritis [11]. Even after treatment, the recurrence rate of PVNS ranges from 14 to 55% [12].

Although the exact pathogenesis of PVNS remains unclear, this disease is presumably closely related to trauma, intraarticular bleeding, and inflammation [13]. Due to the highly variable clinical presentation of PVNS, an accurate diagnosis is extremely difficult to achieve. Advanced imaging modalities, such as magnetic resonance imaging (MRI), are considered the most sensitive approaches for the assessment of suspected PVNS. Soft masses with low signal intensity and “blooming artifact” on MRI scans appear to be pathognomonic of PVNS [1, 14, 15]. However, histopathology remains the gold standard tool to confirm the diagnosis. Surgical treatment for PVNS is considered as the standard treatment for these lesions; conservative treatments, such as radiation therapy, are also available. An in-depth understanding of the extent of the disease is needed to select the most appropriate treatment, as the two types of PVNS require different treatments. Currently, PVNS is classified as diffuse (DPVNS) or localized (LPVNS) [16]. The two types of PVNS can be further categorized as intraarticular or extraarticular. Despite the histological similarity, there are differences between the types in clinical presentation, prognosis, and response to treatment [17, 18].

Here, we describe a patient who had massive intraarticular LPVNS with an extraarticular extension through the lateral patellar retinaculum; the lesion was completely excised in open synovectomy.

## Case presentation

**Fig. 1:**
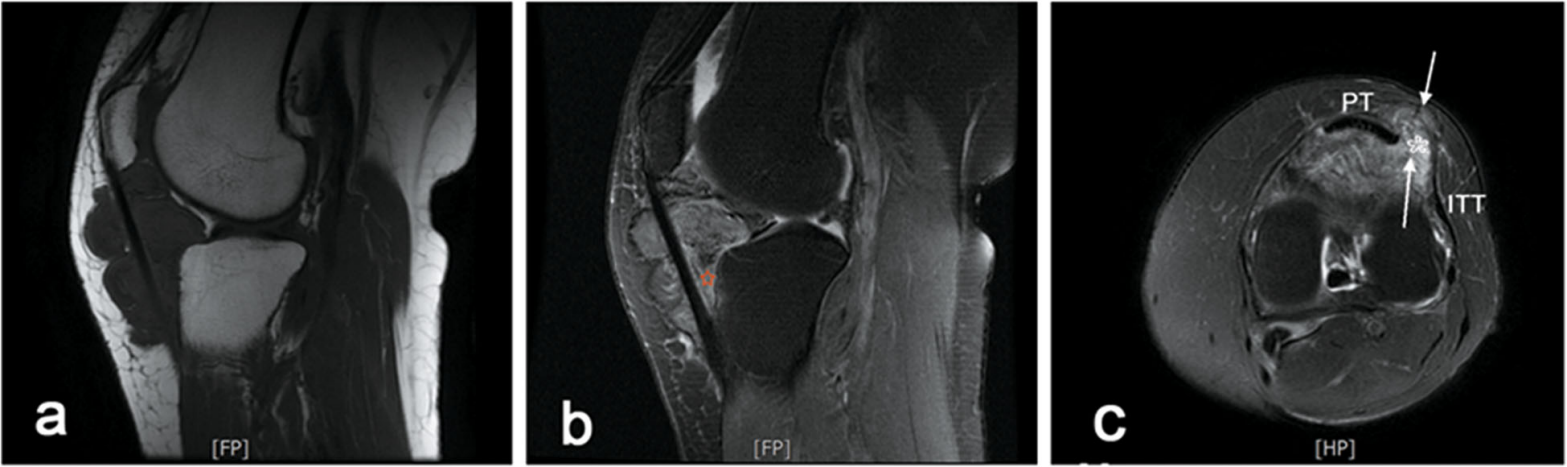
Preoperative MRI scans. **a** LPVNS manifested as a circumscribed and lobulated infrapatellar mass with an extra-articular extension through patellar tendon on sagittal T1-weighted images. **b** The pathognomonic appearance of PVNS, a “blooming artifact” caused by hemosiderin deposition (red pentastar), was observed on sagittal T2-weighted images. **c** Axial images revealed that extra-articular mass developed on both sides of the lateral patellar retinaculum, through a focal defect (asterisk), with a superficial component (arrowhead) and a deep component (arrowhead). ITT: Indicates iliotibial tract; PT: patellar tendon

A 58-year-old woman who did not have a recent history of injury presented to our orthopaedic clinic with a tender palpable soft tissue mass in the anterolateral aspect of the left knee and acute reduced range of motion (ROM). The patient had a history of hyperuricemia for nearly 30 years and experienced knee trauma approximately 20 years ago. She complained of recurrent swelling and pain in the knee, which may have been misdiagnosed as gouty arthritis at the local community hospital because of similar symptoms. The advised treatment based on the symptoms, including colchicine and nonsteroidal anti-inflammatory drugs, partially relieved the patient’s symptoms. The physical examination showed moderate, recurrent swelling of the left knee without surrounding warmth, as well as a tender mass growing slowly in the anteroinferior aspect of the left knee. Because of pain, the patient’s ROM in flexion had decreased considerably (30° – 70° in the left knee, compared with 0° – 110° in the right knee). The results of the laboratory tests for both trioxypurine and infection were within the respective normal limits. The MRI examination of the left knee revealed a massive intraarticular soft tissue lesion (measuring 58 × 32 × 46 mm) that replaced the normal Hoffa fat pad and extended through the lateral patellar retinaculum (Fig. 1a and c). Moreover, the lobulated mass had a well-circumscribed margin and exhibited focal heterogeneous hypointensity representing the blooming artifact from haemosiderin (Fig. 1b), which is regarded as the pathognomonic appearance of PVNS on MRI. Only mild degenerative changes and joint effusion were observed, without tophus or bone erosion.

**Fig. 2:**
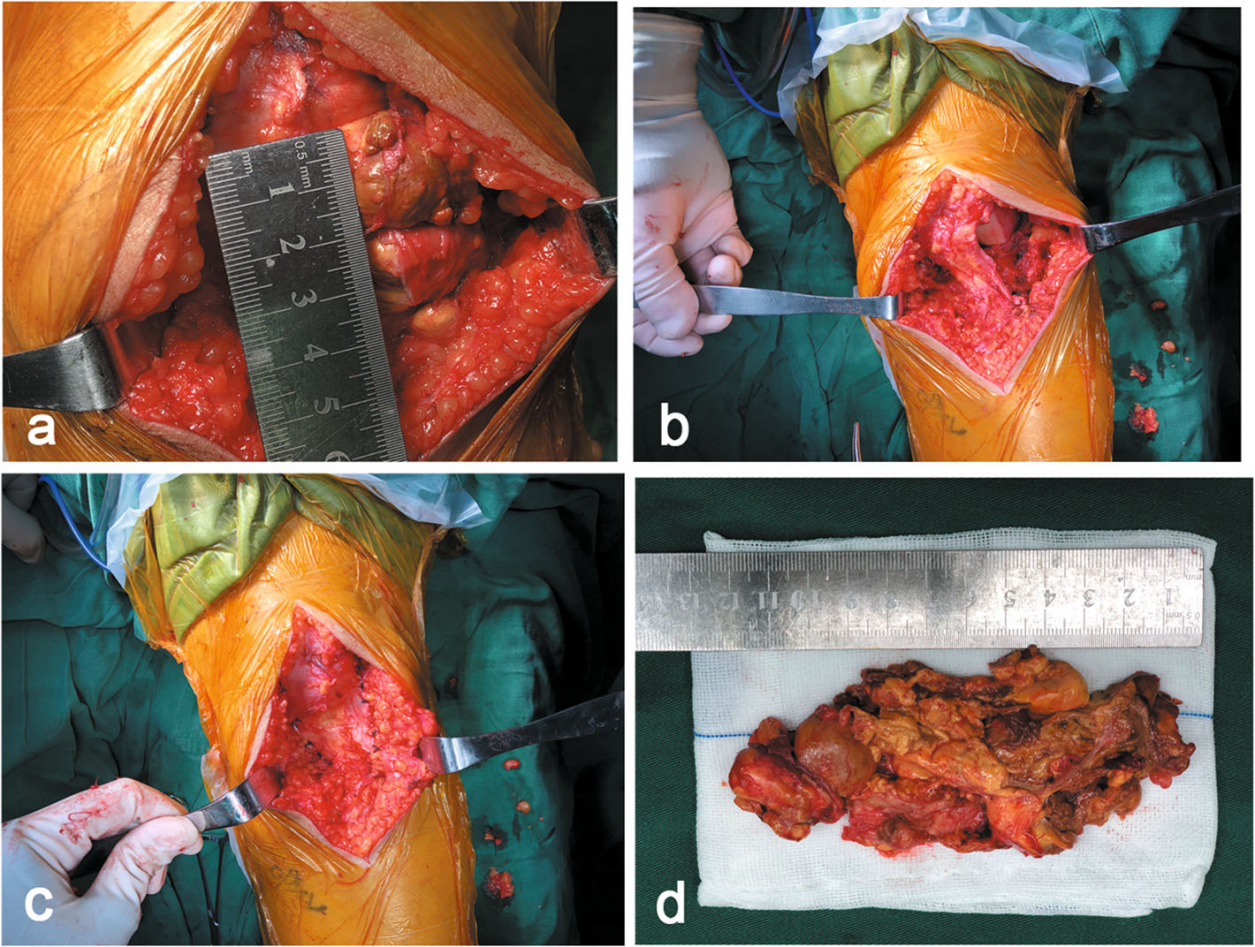
Intraoperative findings. **a** Exposure of the extra-articular mass originating from the lateral patellar retinaculum. (**b**. **c**) As the abnormal synovium was excised completely, the focal defect lateral patellar retinaculum was repaired. **d** The 13 cm median incision was sutured with skin staples

Based on the MRI findings, it was considered impossible to completely remove the mass lesion. Therefore, open excision was selected as the surgical treatment. A 13-cm median incision was made, and synovectomy was performed successfully. A bright brown inflamed lobulated synovium was observed intraoperatively (Fig. 2a). Neither bone erosion nor diffuse soft tissue lesions were observed, suggesting a diagnosis of LPVNS. The final diagnosis was further confirmed by postoperative histological analysis, the results of which were consistent with LPVNS: multinucleated giant cells with no atypical features and an abundance of macrophages loaded with haemosiderin.

**Fig. 3:**
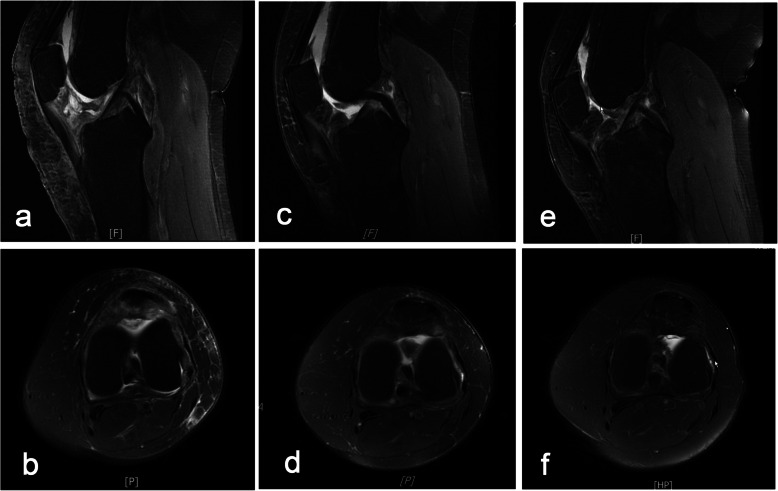
MRI scan at the follow-up. **a**, **b** No recurrence was observed on either the sagittal or axial MRI scans at the 1-month follow-up. **c**-**f** No recurrence was observed at the 6- or 18-month follow-up

Postoperatively, the patient was satisfied with the substantial improvement of knee ROM (5° – 100°). At the 1, 6 and 18-month follow-ups, the patient had no pain and exhibited full restoration of knee ROM, with neither complications nor radiological recurrence (Fig. 3).

## Discussion

Early-stage PVNS is challenging to diagnose because the symptoms of PVNS are atypical. Patients commonly present with symptoms of pain, swelling, instability, reduced ROM, and/or a palpable mass [19]. Other conditions (e.g., various types of synovitis and arthritis, degenerative changes, and soft tissue tumours) need to be excluded before PVNS can be diagnosed. Although the “blooming artifact” on MRI is regarded as the pathognomonic appearance of PVNS, and MRI has therefore become the modality of choice for diagnosis, a definitive diagnosis can only be established histopathologically [7, 20]. Patients may tolerate symptom progression for an extended period of time before seeking treatment [21–23]; it has been reported that the mean duration from the initial onset of PVNS to diagnosis is 2.9 years [2]. Patients between 30 and 50 years of age are more susceptible to developing LPVNS [18]. In the case reported here, the first symptom (a swollen left knee without pain) occurred when the patient was approximately 41 years of age. However, 17 years passed before she was accurately diagnosed with LPVNS. LPVNS is less aggressive than DPVNS, over a long time, the symptoms of the patient remained mild and progressed slowly; in the absence of imaging data, the lesion was not suspected or was misdiagnosed as a process of nonspecific presentation, such as gouty arthritis, rheumatoid arthritis, hemophilia, angiomas, synovial neoplasias, and other inflammatory processes; as well as the history of hyperuricemia, led to a misdiagnosis at the local community hospital. No tophus or bone erosion was observed, either on preoperative MRI scans or intraoperatively. Coincidentally, symptomatic treatment including nonsteroidal anti-inflammatory drugs provided effective symptom relief, which also contributed to the delay in the accurate diagnosis. Ultimately, the diagnostic delay led to a late treatment, while the early diagnosis may contribute to the better clinical evaluation, especially in improving the postoperative discomfort and limited range of motion, MRI examination is capable of showing the typical characteristic of PVNS and plays a crucial role in the initial diagnosis. Moreover, preoperative MRI is used to identify the intra- and extraarticular lesion which is conducive to avoid incomplete excision of the tumor leading to recurrence. Therefore, before PVNS is confirmed, we recommend that MRI examinations are performed in all patients suspected of having PVNS with repeated joint swelling, pain, joint mobility limitation and joint effusion, including those who have a history of hyperuricemia.

In the knee, LPVNS typically presents as a focal mass, frequently in the anterior compartment [18]. The mass occasionally extrudes into the articular cavity and can vary in diameter from a few millimetres to 3 cm [20, 24]. The mechanical symptoms of LPVNS vary in relation to the lesion size and location [25]. Although LPVNS tends to exhibit a more slowly destructive course, focal bone destruction has been observed in some patients. In the patient in our study, the lesion features were notable. The intra- and extraarticular mass, measuring 125 × 49 mm (in vitro, Fig. 2d), is the largest reported thus far; this large size may be related to the relatively long course of the disease. The mass was located in the anteroinferior joint space with extraarticular extension through the lateral patellar retinaculum (Fig. 2a), which may have been associated with the focal defect of the lateral patellar retinaculum [26]; this condition was particularly likely to occur in the patient in our study, as she experienced localized left knee trauma 20 years ago. However, only mild degenerative changes and joint effusion were observed. The anteroinferior joint space is relatively large and the intraarticular mass can herniate through the focal defect of the lateral patellar retinaculum during knee flexion. Since no similar PVNS cases were found in the literature, our article is - to our knowledge - the first to report a case of massive intraarticular LPVNS.

Currently, there is no standard method for nonsurgically observing LPVNS. The eradication of all aberrant synovial tissue in symptomatic patients is considered a suitable treatment option for LPVNS, as it effectively relieves pain and prevents local recurrence. Nevertheless, the optimal type of resection and whether adjuvant therapy should be used remains controversial [27]. Open synovectomy is routinely performed for extraarticular extension of LPVNS when full excision cannot be achieved with an arthroscope [20, 28]. However, open excision may lead to postoperative muscle weakness and an increased risk of osteoarthritic changes. With advances in arthroscopy, all-arthroscopic total synovectomy has been shown to be equally effective [20, 29–31]. In addition, the combination of the two techniques using a posterior open and anterior arthroscopic approach may be ideal when commonly used portals cannot be used to completely excise the posterior abnormal synovial [32]. In recent years, some research has suggested that it is necessary to excise some otherwise normal-appearing fat and areolar tissue with the synovium in cases of recurrence [33]. In general, the appropriate extent of excision should be determined on the basis of the type of PVNS, the presence of extraarticular extensions, and the experience of the surgeon. In addition, conventional radiotherapy, as well as radiosynoviorthesis, is considered for patients with large and even recurrent diffuse forms of the disease [34]. Preoperatively, although the patient had been recommended to receive radiosynoviorthesis 6–8 weeks after surgery, she declined to accept the adjuvant therapy because of the concerns about the potential side effects of the radiopharmacon, Fortunately, both intraoperative findings and the postoperative MRI ensured that no residual PVNS was found. So far, the complete surgical excision without radiation therapy is satisfactory in the present case. Nevertheless, PVNS still has a certain rate of recurrence; in some cases, recurrence occurs even after 16 years postoperatively [35]. The diffuse form of the disease, incomplete resection, the lesions being located in locations difficult to access, the surgeon having limited experience and skills, and the administration of adjuvant therapy after surgery have been identified as the major risk factors for recurrence [27]. Approximately two-thirds of local recurrences are diagnosed within postoperative 2 years, while less than 10% of recurrences are found after 3 years, and it is recommended that follow-up MRI scans are performed every 6 months during the first 3 years to detect local recurrences effectively [36]. In this patient, we successfully performed open complete excision. Symptom relief was achieved, and no recurrence was observed during the subsequent follow-up (Fig. 3); however, the long-term curative effect remains to be assessed.

## Conclusions

To reduce the occurrence of misdiagnosis, MRI examinations are recommended for all patients suspected of having PVNS, including those who have a history of hyperuricemia. Early diagnosis with prompt surgical treatment may lead to favourable recovery.

## Data Availability

The datasets used and analysed during the current study are available from the corresponding author on reasonable request.

## Abbreviations

PVNS: Pigmented villonodular synovitis
DPVNS: Diffuse pigmented villonodular synovitis
LPVNS: Localized pigmented villonodular synovitis
ROM: Range of motion
MRI: Magnetic Resonance Imaging
